# Supplementary material for: Pervasive tissue-, genetic background-, and allele-specific gene expression effects in Drosophila melanogaster
Source: PLoS Genet. 2024 Aug 23;20(8):e1011257. doi: 10.1371/journal.pgen.1011257 (PMC11376557; doi:10.1371/journal.pgen.1011257)
Supplement: S1 Fig — A) Expression and B) dominance (h) divergence among genotypes within the midgut (MG), hindgut (HG), and Malpighian tubule (MT) versus divergence between the same genotype among tissues (across). C) Expression and D) dominance (h) divergence within the same genotype among tissues. A–C) Significance was assessed with a t-test. D) Significance was not assessed due to the low number of comparisons. Bonferroni-corrected P values are shown. * P < 0.05, ** P < 5 x 10^−5^, *** P < 10−14, ns not significant, nt not tested. (PDF) [file pgen.1011257.s001.pdf]

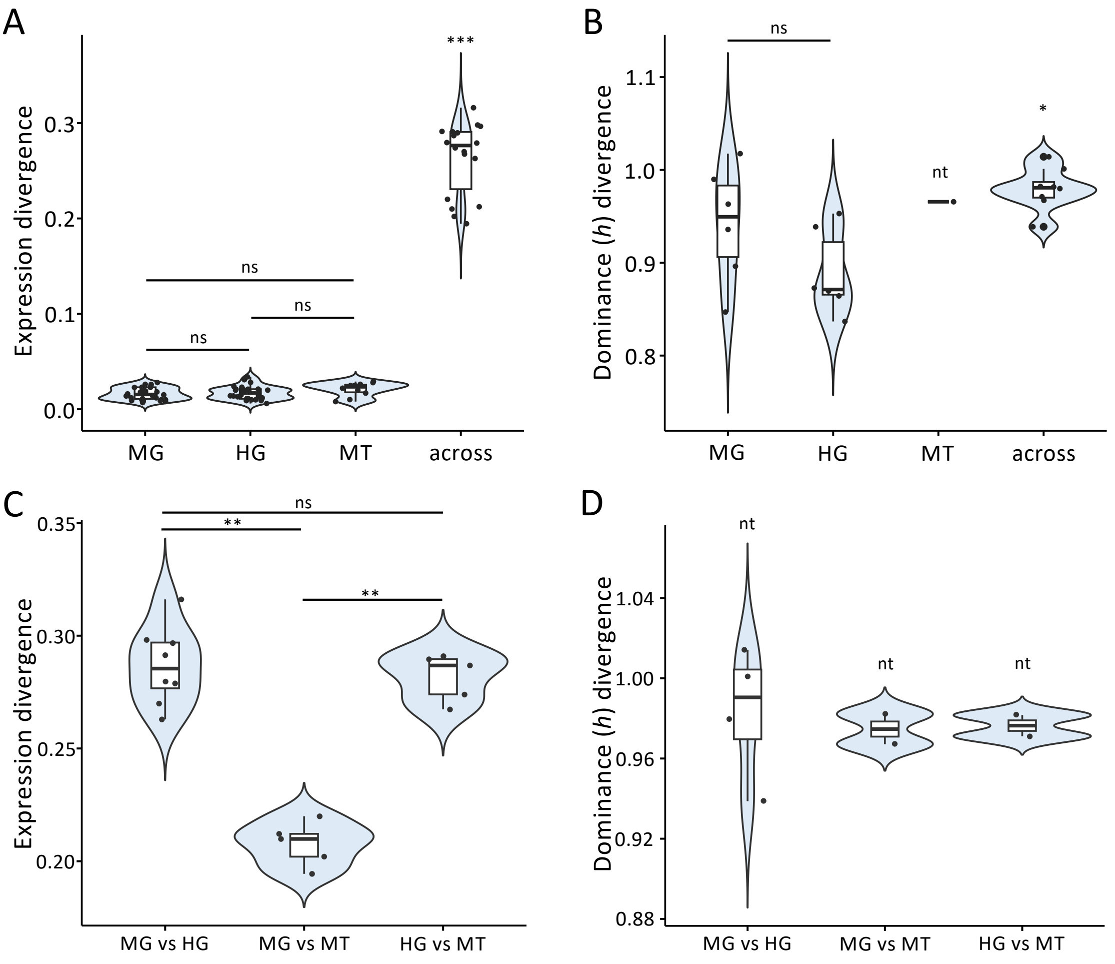


**S1 Fig. Expression and dominance (*h*) divergence within and among tissues.** A) Expression and B) dominance (*h*) divergence among genotypes within the midgut (MG), hindgut (HG), and Malpighian tubule (MT) versus divergence between the same genotype among tissues (across). C) Expression and D) dominance (*h*) divergence within the same genotype among tissues. A–C) Significance was assessed with a *t*-test. D) Significance was not assessed due to the low number of comparisons. Bonferroni-corrected *P* values are shown. * $P < 0.05$, ** $P < 5 \times 10^{-5}$, *** $P < 10^{-14}$, ns not significant, nt not tested.
