# Supplementary material for: Pervasive tissue-, genetic background-, and allele-specific gene expression effects in Drosophila melanogaster
Source: PLoS Genet. 2024 Aug 23;20(8):e1011257. doi: 10.1371/journal.pgen.1011257 (PMC11376557; doi:10.1371/journal.pgen.1011257)
Supplement: S2 Fig — The total number of differentially expressed (DE) genes between genotypes within the A) hindgut (HG), B) midgut (MG), and C) Malpighian tubule (MT) are shown above the diagonal, while expression divergence (as measured by ρ subtracted from one) between genotypes is shown below the diagonal. Analysis was performed in each tissue individually. The numbers of genes that could be included in the analysis for each tissue were 8,209 in the hindgut, 7,684 in the midgut, and 7,675 in the Malpighian tubule. (PDF) [file pgen.1011257.s002.pdf]

A

HG DE genes

HG divergence (1 - ρ)

	SU26	SU58	ZI197	ZI418	SU26xZI197	SU26xZI418	SU58xZI197	SU58xZI418
**SU26**		2137	3068	3206	1221	1092	2178	2365
**SU58**	0.029		2628	2708	1851	2070	1397	1099
**ZI197**	0.042	0.029		2926	1405	2938	1408	2841
**ZI418**	0.039	0.026	0.029		2679	1645	3180	1432
**SU26xZI197**	0.018	0.022	0.014	0.028		963	479	1738
**SU26xZI418**	0.014	0.024	0.035	0.019	0.012		1863	880
**SU58xZI197**	0.030	0.016	0.010	0.029	0.007	0.023		1529
**SU58xZI418**	0.029	0.012	0.027	0.014	0.020	0.016	0.015	

B

MG DE genes

MG divergence (1 - ρ)

	SU26	SU58	ZI197	ZI418	SU26xZI197	SU26xZI418	SU58xZI197	SU58xZI418
**SU26**		1950	2115	2607	1092	1523	1584	1733
**SU58**	0.026		1897	2153	1133	1426	1085	503
**ZI197**	0.035	0.031		2083	799	2036	894	1349
**ZI418**	0.038	0.033	0.029		2345	1176	2069	773
**SU26xZI197**	0.016	0.020	0.013	0.027		555	457	630
**SU26xZI418**	0.018	0.023	0.028	0.016	0.011		1281	352
**SU58xZI197**	0.025	0.017	0.013	0.027	0.009	0.018		831
**SU58xZI418**	0.023	0.011	0.022	0.014	0.012	0.008	0.012	

C

MT DE genes

MT divergence (1 - ρ)

	SU26	SU58	ZI418	SU26xZI418	SU58xZI418
**SU26**		1780	2184	2163	1683
**SU58**	0.027		1482	1312	348
**ZI418**	0.033	0.032		729	466
**SU26xZI418**	0.032	0.031	0.024		462
**SU58xZI418**	0.025	0.010	0.013	0.019	

S2 Fig. Differential expression and divergence within tissues. The total number of differentially expressed (DE) genes between genotypes within the A) hindgut (HG), B) midgut (MG), and C) Malpighian tubule (MT) are shown above the diagonal, while expression divergence (as measured by ρ subtracted from one) between genotypes is shown below the diagonal. Analysis was performed in each tissue individually. The numbers of genes that could be included in the analysis for each tissue were 8,209 in the hindgut, 7,684 in the midgut, and 7,675 in the Malpighian tubule.
