# Supplementary material for: Pervasive tissue-, genetic background-, and allele-specific gene expression effects in Drosophila melanogaster
Source: PLoS Genet. 2024 Aug 23;20(8):e1011257. doi: 10.1371/journal.pgen.1011257 (PMC11376557; doi:10.1371/journal.pgen.1011257)
Supplement: S3 Fig — Upset plots showing unique and overlapping genes within the hindgut (circles), midgut (triangles), and Malpighian tubule (squares) in the A,C,E) SU26xZI418 or B,D,F) SU58xZI418 backgrounds. Horizontal bars represent the total number (num.) of genes in a tissue and inheritance category combination. Vertical bars represent the number of genes in an intersection class. A filled circle underneath a vertical bar indicates that a tissue and inheritance category combination is included in an intersection class. A single filled circle represents an intersection class containing only genes unique to a single tissue and inheritance category combination. Filled circles connected by a line indicate that multiple tissue and inheritance category combinations are included in an intersection class. Genes categorized into A,B) basic expression inheritance (inherit.), i.e. P1 dominant (P1 dom.), P2 dominant (P2 dom.), and additive (add.), C,D) misexpression (misexpress.), and E,F) similar categories are shown. (PDF) [file pgen.1011257.s003.pdf]

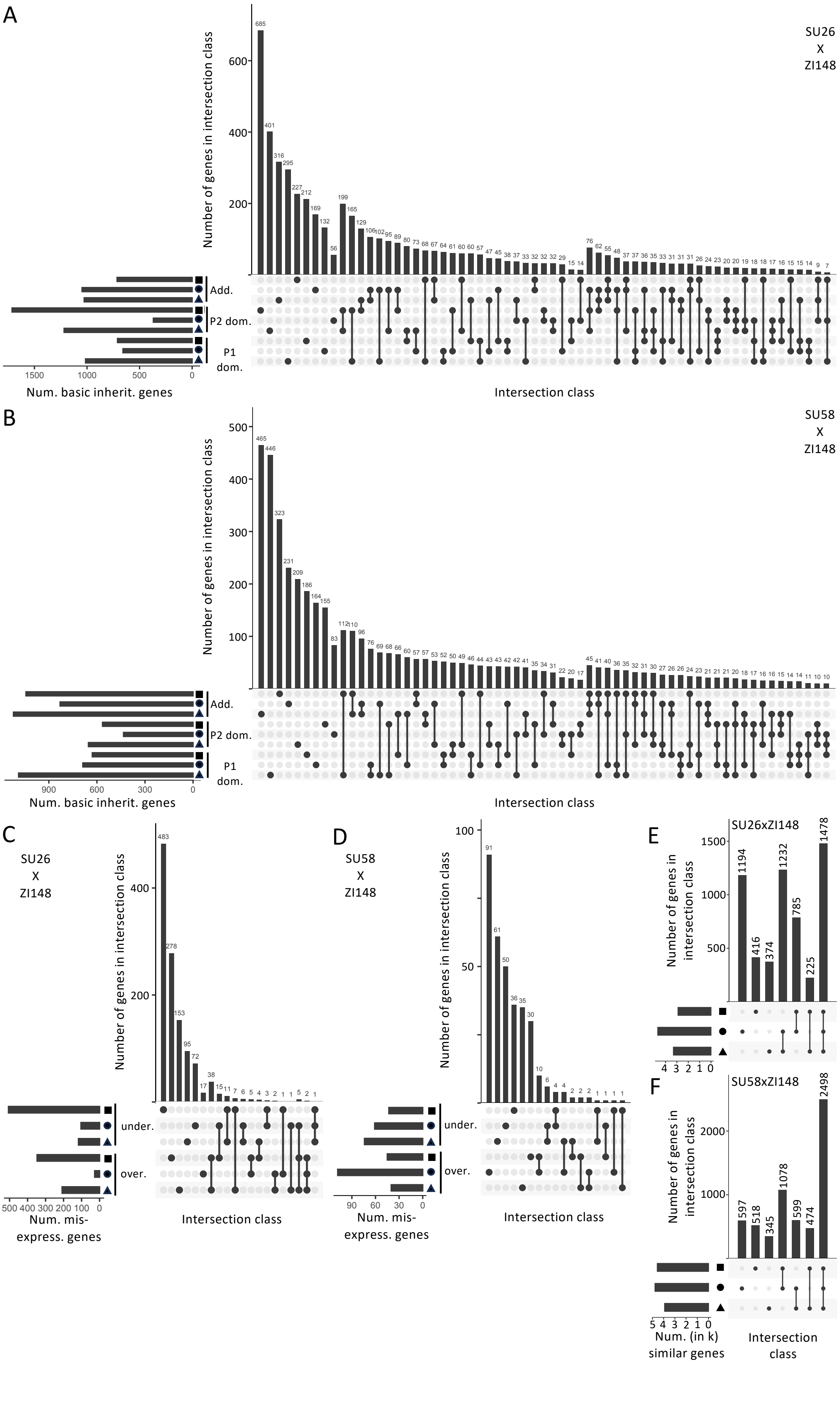
A
SU26
X
ZI148
Number of genes in intersection class
685 401 316 295 227 212 169 132 56 199 165 129 106 102 95 89 80 73 68 67 64 61 60 60 57 47 45 38 37 33 32 32 32 29 15 14 76 62 55 48 37 37 36 35 33 31 31 31 26 24 23 20 20 19 18 18 17 16 15 15 14 9 7
Add.
P2 dom.
P1 dom.
Num. basic inherit. genes
Intersection class
B
SU58
X
ZI148
Number of genes in intersection class
465 446 323 231 209 186 164 155 83 112 110 96 76 69 68 66 60 57 57 53 52 50 49 46 44 43 43 42 42 41 35 34 31 22 20 17 45 41 40 36 35 32 31 30 27 26 26 24 23 21 21 21 20 18 17 16 16 15 14 14 11 10 10
Add.
P2 dom.
P1 dom.
Num. basic inherit. genes
Intersection class
C
SU26
X
ZI148
Number of genes in intersection class
483 278 153 95 72 17 38 15 11 7 6 5 4 3 2 1 1 5 2 1
under.
over.
Num. mis-express. genes
Intersection class
D
SU58
X
ZI148
Number of genes in intersection class
91 61 50 36 35 30 10 6 4 4 2 2 2 1 1 1 1
under.
over.
Num. mis-express. genes
Intersection class
E
SU26xZI148
Number of genes in intersection class
1194 416 374 1232 785 225 1478
F
SU58xZI148
Number of genes in intersection class
597 518 345 1078 599 474 2498
Num. (in k) similar genes
Intersection class

S3 Fig. Mode of expression Inheritance in SU26xZI418 and SU58xZI418 backgrounds. Upset plots showing unique and overlapping genes within the hindgut (circles), midgut (triangles), and Malpighian tubule (squares) in the A,C,E) SU26xZI418 or B,D,F) SU58xZI418 backgrounds. Horizontal bars represent the total number (num.) of genes in a tissue and inheritance category combination. Vertical bars represent the number of genes in an intersection class. A filled circle underneath a vertical bar indicates that a tissue and inheritance category combination is included in an intersection class. A single filled circle represents an intersection class containing only genes unique to a single tissue and inheritance category combination. Filled circles connected by a line indicate that multiple tissue and inheritance category combinations are included in an intersection class. Genes categorized into A,B) basic expression inheritance (inherit.), i.e. P1 dominant (P1 dom.), P2 dominant (P2 dom.), and additive (add.), C,D) misexpression (misexpress.), and E,F) similar categories are shown.
