# Supplementary material for: Pervasive tissue-, genetic background-, and allele-specific gene expression effects in Drosophila melanogaster
Source: PLoS Genet. 2024 Aug 23;20(8):e1011257. doi: 10.1371/journal.pgen.1011257 (PMC11376557; doi:10.1371/journal.pgen.1011257)
Supplement: S4 Fig — Upset plots showing unique and overlapping genes within the hindgut (circles) and midgut (triangles) in the A,B,E) SU26xZI197 or C,D,F) SU58xZI197 backgrounds. Horizontal bars represent the total number (num.) of genes in a tissue and inheritance category combination. Vertical bars represent the number of genes in an intersection class. A filled circle underneath a vertical bar indicates that a tissue and inheritance category combination is included in an intersection class. A single filled circle represents an intersection class containing only genes unique to a single tissue and inheritance category combination. Filled circles connected by a line indicate that multiple tissue and inheritance category combinations are included in an intersection class. Genes categorized into A,C) similar, B,D) basic expression inheritance (inherit.), i.e. P1 dominant (P1 dom.), P2 dominant (P2 dom.), and additive (add.), and E,F) misexpression (mis-express.) categories are shown. (PDF) [file pgen.1011257.s004.pdf]

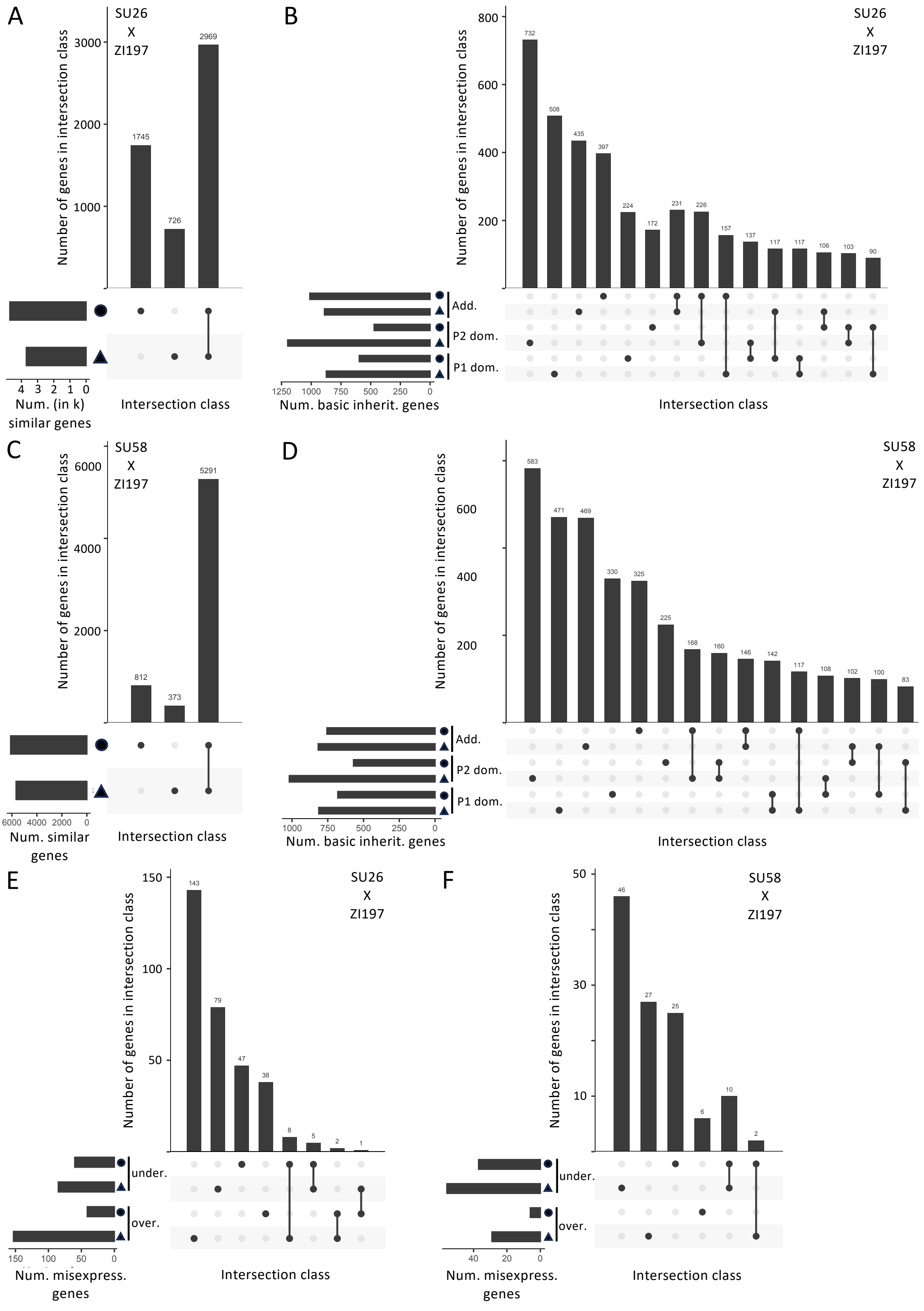

S4 Fig. Mode of expression inheritance in SU26xZI197 and SU58xZI197 backgrounds. Upset plots showing unique and overlapping genes within the hindgut (circles) and midgut

(triangles) in the A,B,E) SU26xZI197 or C,D,F) SU58xZI197 backgrounds. Horizontal bars represent the total number (num.) of genes in a tissue and inheritance category combination. Vertical bars represent the number of genes in an intersection class. A filled circle underneath a vertical bar indicates that a tissue and inheritance category combination is included in an intersection class. A single filled circle represents an intersection class containing only genes unique to a single tissue and inheritance category combination. Filled circles connected by a line indicate that multiple tissue and inheritance category combinations are included in an intersection class. Genes categorized into A,C) similar, B,D) basic expression inheritance (inherit.), i.e. P1 dominant (P1 dom.), P2 dominant (P2 dom.), and additive (add.), and E,F) misexpression (mis-express.) categories are shown.
