# Supplementary material for: Pervasive tissue-, genetic background-, and allele-specific gene expression effects in Drosophila melanogaster
Source: PLoS Genet. 2024 Aug 23;20(8):e1011257. doi: 10.1371/journal.pgen.1011257 (PMC11376557; doi:10.1371/journal.pgen.1011257)
Supplement: S5 Fig — Upset plots showing unique and overlapping genes with non-ambiguous regulatory divergence in the hindgut (circles), midgut (triangles), and Malpighian tubule (squares) in the A) SU26xZI418 or B) SU58xZI418 backgrounds. Horizontal bars represent the total number of genes in a tissue and regulatory category combination. Vertical bars represent the number of genes in an intersection class. A filled circle underneath a vertical bar indicates that a tissue and inheritance category combination is included in an intersection class. A single filled circle represents an intersection class containing only genes unique to a single tissue and regulatory category combination. Filled circles connected by a line indicate that multiple tissue and regulatory category combinations are included in an intersection class. (PDF) [file pgen.1011257.s005.pdf]

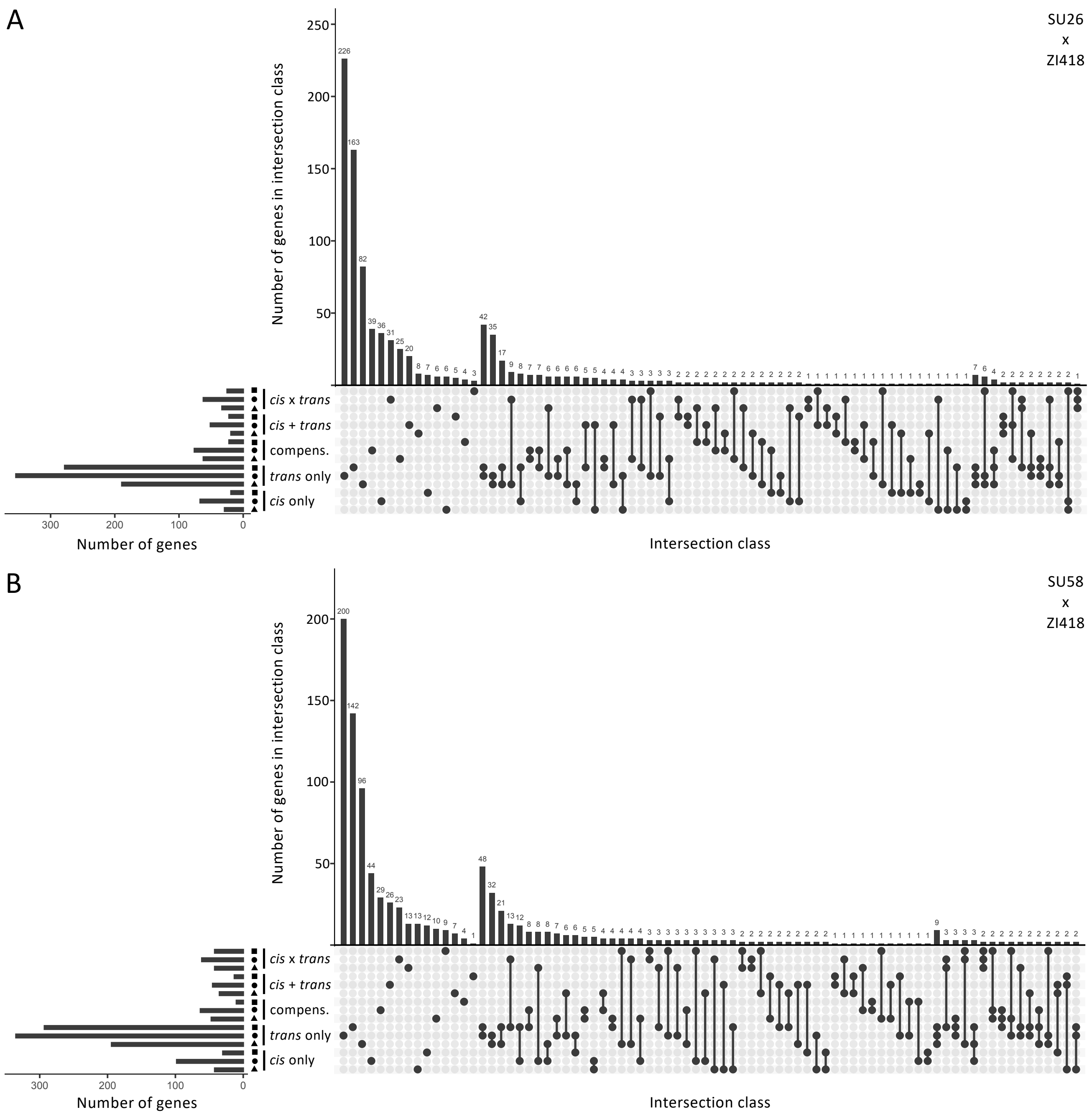

S5 Fig. Genetic basis of expression inheritance in SU26xZI418 and SU58xZI418 backgrounds. Upset plots showing unique and overlapping genes with non-ambiguous regulatory divergence in the hindgut (circles), midgut (triangles), and Malpighian tubule (squares) in the A) SU26xZI418 or B) SU58xZI418 backgrounds. Horizontal bars represent the total number of genes in a tissue and regulatory category combination. Vertical bars represent the number of genes in an intersection class. A filled circle underneath a vertical bar indicates that a tissue and inheritance category combination is included in an intersection class. A single filled circle represents an intersection class containing only genes unique to a single tissue and regulatory category combination. Filled circles connected by a line indicate that multiple tissue and regulatory category combinations are included in an intersection class.
