# Supplementary material for: Pervasive tissue-, genetic background-, and allele-specific gene expression effects in Drosophila melanogaster
Source: PLoS Genet. 2024 Aug 23;20(8):e1011257. doi: 10.1371/journal.pgen.1011257 (PMC11376557; doi:10.1371/journal.pgen.1011257)
Supplement: S7 Fig — Shown are A,B) unique and C,D) overlapping genes in each regulatory category. Shown are A) the number of genes unique to each tissue within each regulatory category and genetic background, B) the number of genes unique to each genetic background and tissue within each regulatory category, C) the number of genes in each regulatory category detected in all examined tissues for each genetic background, and D) the number of genes in each regulatory category detected in all genetic backgrounds for each tissue. Asterisks (*) indicate comparisons using only C) two tissues or D) two genetic backgrounds. (PDF) [file pgen.1011257.s007.pdf]

A

	SU58xZI418			SU58xZI197		SU26xZI418			SU26xZI197	
	Hindguts	Midguts	Malp Tub	Hindguts	Midguts	Hindguts	Midguts	Malp Tub	Hindguts	Midguts
conserved	260	248	633	54	59	181	324	331	161	153
all cis	70	29	27	17	4	40	15	16	56	12
all trans	134	95	201	190	165	146	67	186	94	79
compensatory	44	34	8	3	10	50	49	20	66	42
cis + trans	25	23	10	17	23	24	10	19	48	23
cis x trans	30	26	31	29	18	30	13	14	74	27
ambiguous	112	86	342	590	232	121	62	666	129	71

B

	SU58xZI418			SU58xZI197		SU26xZI418			SU26xZI197	
	Hindguts	Midguts	Malp Tub	Hindguts	Midguts	Hindguts	Midguts	Malp Tub	Hindguts	Midguts
conserved	173	165	522	52	57	140	269	280	148	140
all cis	69	27	26	17	4	39	14	16	54	10
all trans	115	77	179	171	146	129	58	172	89	74
compensatory	39	30	7	3	10	46	45	18	62	38
cis + trans	23	23	8	15	21	24	9	18	48	23
cis x trans	26	23	28	26	15	29	12	14	69	22
ambiguous	92	68	312	533	175	85	34	615	122	64

C

	SU58 x ZI418	SU26 x ZI418	SU58 x ZI197*	SU26 x ZI197*
conserved	432	487	110	443
all cis	2	1	0	8
all trans	9	7	80	38
compensatory	0	0	0	7
cis + trans	0	2	6	9
cis x trans	0	1	13	15
ambiguous	134	202	1461	742

D

	Hindguts	Midguts	Malp Tub*
conserved	216	70	1215
all cis	3	1	3
all trans	14	18	92
compensatory	0	1	2
cis + trans	0	0	3
cis x trans	5	5	11
ambiguous	116	650	267

S7 Fig. Genetic basis of expression inheritance across examined tissues and backgrounds. Shown are A,B) unique and C,D) overlapping genes in each regulatory category. Shown are A) the number of genes unique to each tissue within each regulatory category and genetic background, B) the number of genes unique to each genetic background and tissue within each regulatory category, C) the number of genes in each regulatory category detected in all examined tissues for each genetic background, and D) the number of genes in each regulatory category detected in all genetic backgrounds for each tissue. Asterisks (*) indicate comparisons using only C) two tissues or D) two genetic backgrounds.
