# Supplementary material for: Pervasive tissue-, genetic background-, and allele-specific gene expression effects in Drosophila melanogaster
Source: PLoS Genet. 2024 Aug 23;20(8):e1011257. doi: 10.1371/journal.pgen.1011257 (PMC11376557; doi:10.1371/journal.pgen.1011257)
Supplement: S8 Fig — A) Dominance and B) magnitude of dominance h for genes categorized as cis-only (c, light) and trans-only (t, dark) in each background and tissue. Significance was assessed with a t-test. Bonferroni-corrected P values are shown in grey. *** P < 0.005, ** P < 0.01, * P < 0.05, ms P marginally significant after multiple test correction (P < 0.1), ns P not significant after multiple test correction. (PDF) [file pgen.1011257.s008.pdf]

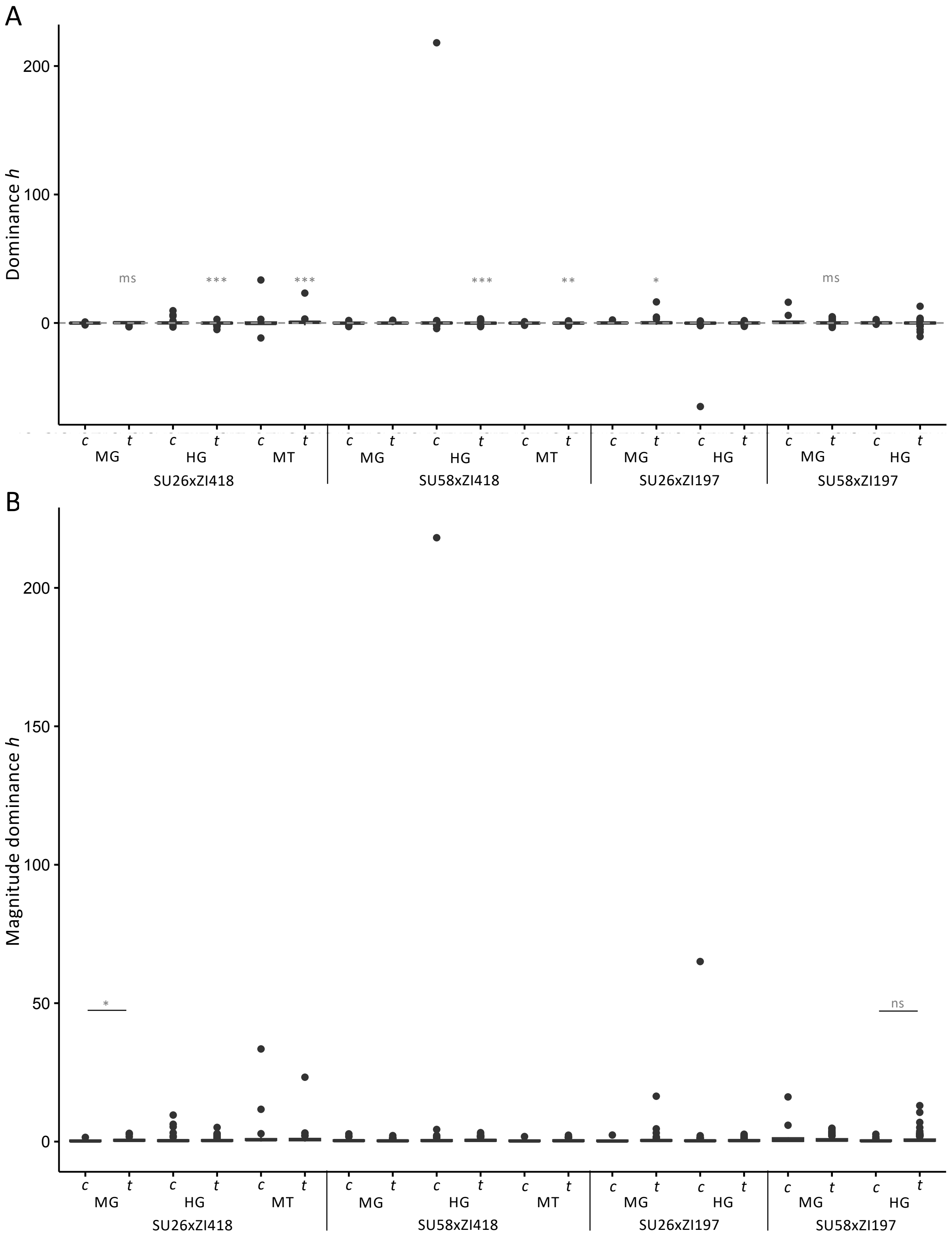

S8 Fig. All dominance in *cis*-only versus *trans*-only genes. A) Dominance and B) magnitude of dominance *h* for genes categorized as *cis*-only (*c*, light) and *trans*-only (*t*, dark) in each background and tissue. Significance was assessed with a *t*-test. Bonferroni-corrected *P* values are shown in grey. *** *P* < 0.005, ** *P* < 0.01, * *P* < 0.05, ms *P* marginally significant after multiple test correction (*P* < 0.1), ns *P* not significant after multiple test correction.
