# Supplementary material for: Pervasive tissue-, genetic background-, and allele-specific gene expression effects in Drosophila melanogaster
Source: PLoS Genet. 2024 Aug 23;20(8):e1011257. doi: 10.1371/journal.pgen.1011257 (PMC11376557; doi:10.1371/journal.pgen.1011257)
Supplement: S9 Fig — Colored sections of each bar show bacterial genera with a relative abundance superior to 5% in each sample. The remaining genera are compiled in the “Others” category. (PDF) [file pgen.1011257.s009.pdf]

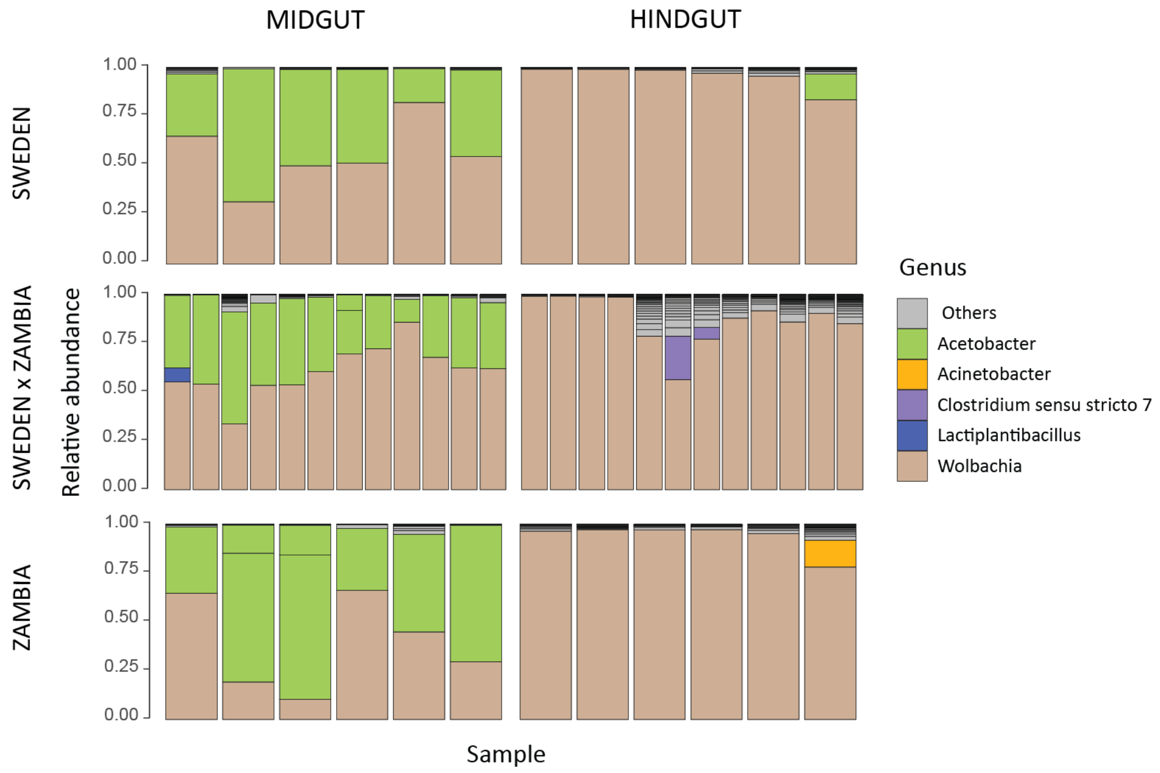

S9 Fig. Composition of the bacterial communities in the midgut and hindgut of each genotype, including *Wolbachia* ASVs. Colored sections of each bar show bacterial genera with a relative abundance superior to 5% in each sample. The remaining genera are compiled in the "Others" category.
