# Supplementary material for: Pervasive tissue-, genetic background-, and allele-specific gene expression effects in Drosophila melanogaster
Source: PLoS Genet. 2024 Aug 23;20(8):e1011257. doi: 10.1371/journal.pgen.1011257 (PMC11376557; doi:10.1371/journal.pgen.1011257)
Supplement: S10 Fig — The legend indicates that replicates of each genotype share the same color, while shape indicates tissue. (PDF) [file pgen.1011257.s010.pdf]

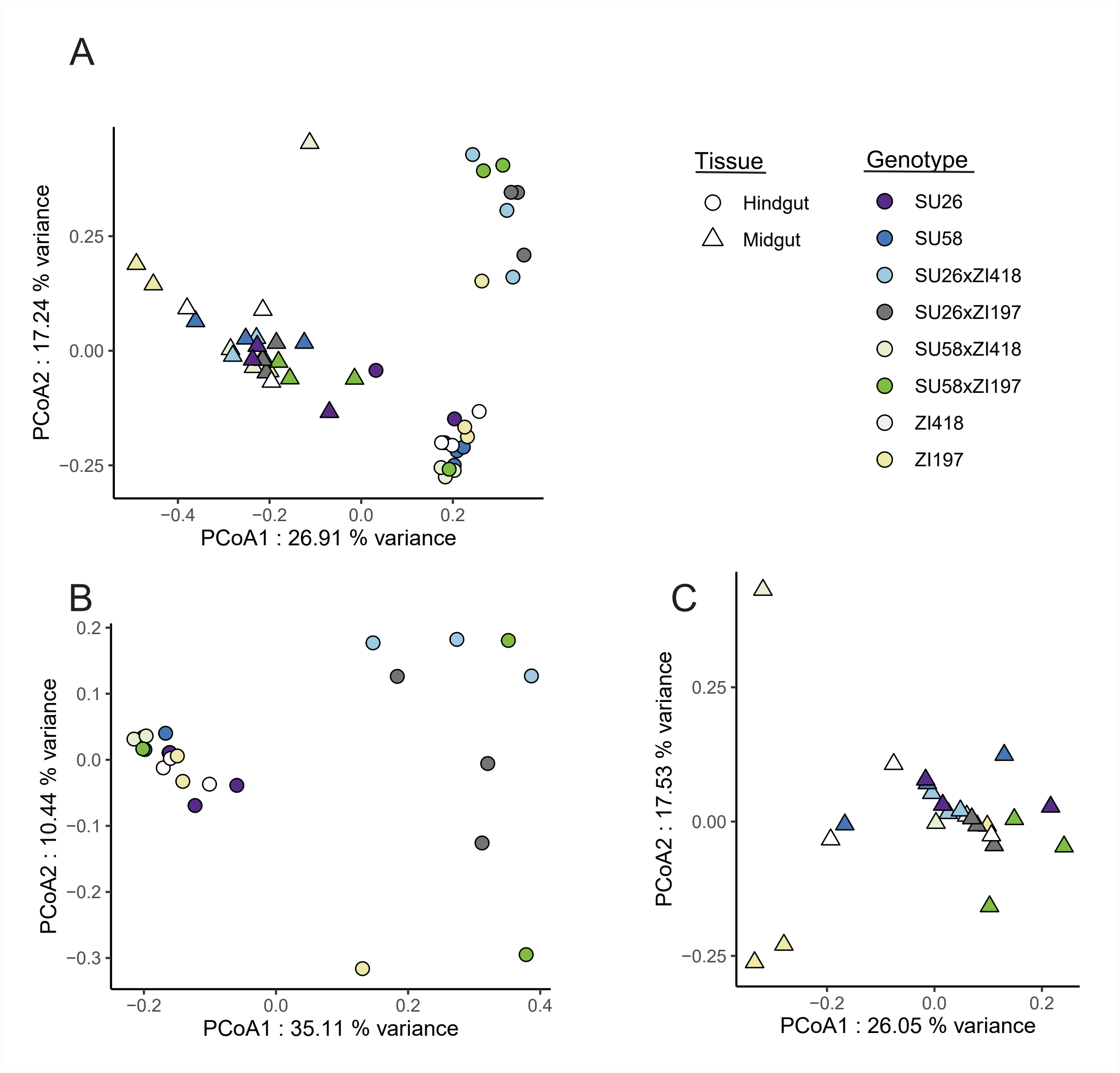

S10 Fig. Principal coordinate analysis of bacterial communities in A) both midgut and hindgut samples, B) hindgut, and C) midgut, including *Wolbachia* ASVs. The legend indicates that replicates of each genotype share the same color, while shape indicates tissue.
