# Supplementary material for: Pervasive tissue-, genetic background-, and allele-specific gene expression effects in Drosophila melanogaster
Source: PLoS Genet. 2024 Aug 23;20(8):e1011257. doi: 10.1371/journal.pgen.1011257 (PMC11376557; doi:10.1371/journal.pgen.1011257)
Supplement: S11 Fig — * indicates significant differences of the Shannon index between groups (lmer, P < 0.05). (PDF) [file pgen.1011257.s011.pdf]

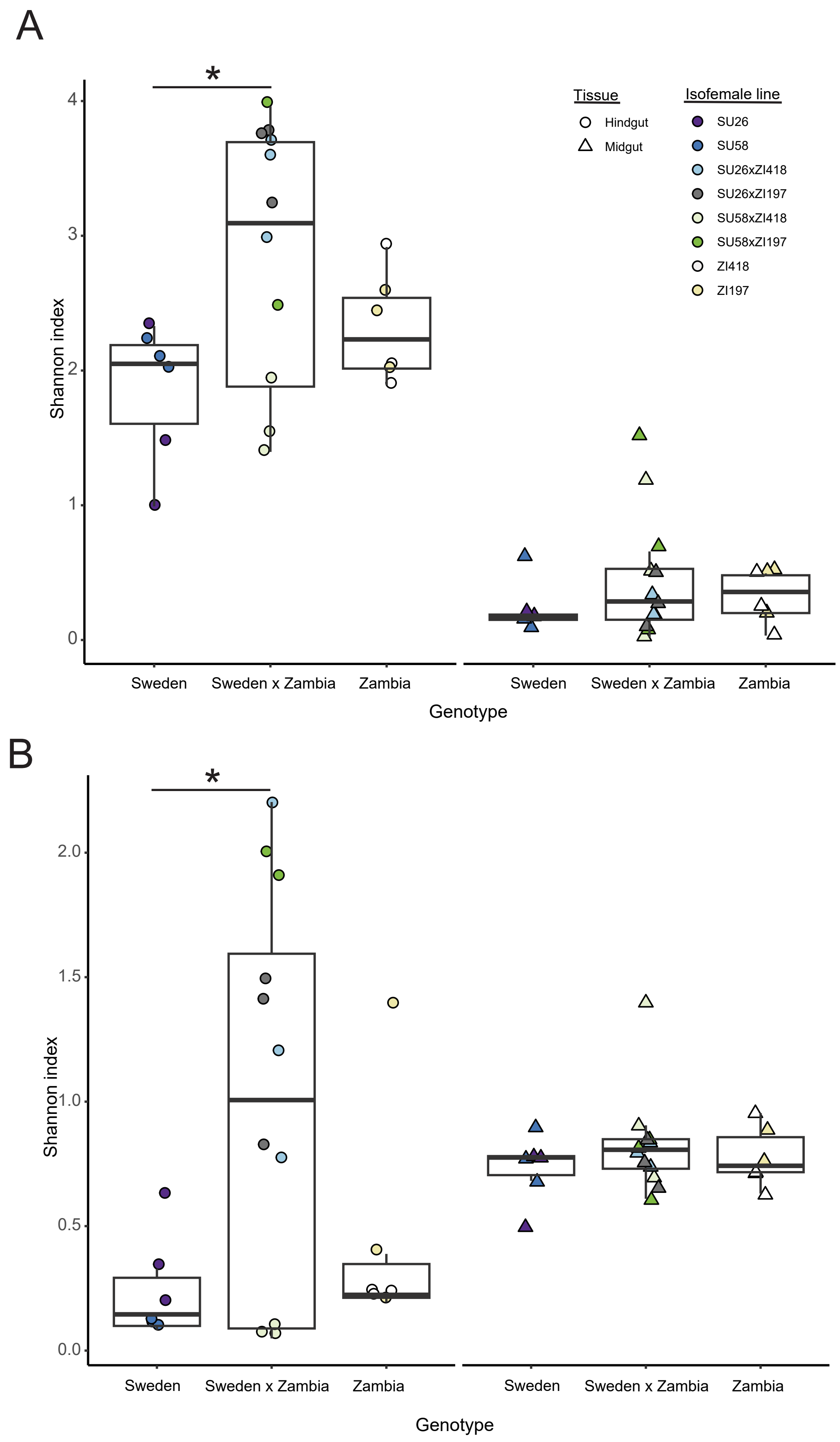
A
*
Tissue
Hindgut
Midgut
Isofemale line
SU26
SU58
SU26xZI418
SU26xZI197
SU58xZI418
SU58xZI197
ZI418
ZI197
Shannon index
0
1
2
3
4
Sweden
Sweden x Zambia
Zambia
Sweden
Sweden x Zambia
Zambia
Genotype
B
*
Shannon index
0.0
0.5
1.0
1.5
2.0
Sweden
Sweden x Zambia
Zambia
Sweden
Sweden x Zambia
Zambia
Genotype

**S11 Fig. Shannon diversity index of the bacterial community in the midgut and hindgut excluding (A) or including (B) *Wolbachia* ASVs.** * indicates significant differences of the Shannon index between groups (lmer, $P < 0.05$).
