# Supplementary material for: Pervasive tissue-, genetic background-, and allele-specific gene expression effects in Drosophila melanogaster
Source: PLoS Genet. 2024 Aug 23;20(8):e1011257. doi: 10.1371/journal.pgen.1011257 (PMC11376557; doi:10.1371/journal.pgen.1011257)
Supplement: Attachment — Submitted filename: Response_to_Reviewers.pdf [file pgen.1011257.s034.pdf]

Dear Dr. Mackay and Dr. Williams,

We thank you and the reviewers for your/their time and feedback. We have revised our manuscript to address your and the reviewer comments. Briefly, among other changes, we have now included an examination of the overlap in differentially expressed genes among genotypes and tissues, the effect of genotype on *Wolbachia* abundance, as well as the effect of sex bias and inversion status on regulatory variation, the results of which can be found in the new Supplemental Tables S1, S8, S9, and S14. All other Supplementary Tables have been renumbered accordingly. Additionally, we have split our S1 Data file into two files (S1 and S2 Data) and added our sex bias estimates to the S2 Data file. All other Supplementary Data files have been renumbered accordingly. We also fixed several typos and rephrased text as well as revised Figures 1, 4, 7, S10, and S11, which we hope will improve reader comprehension.

Sincerely,
Amanda Glaser-Schmitt, Marion Lemoine, Martin Kaltenpoth, and John Parsch

Response to Reviewers

Reviewer #1: The authors examine gene expression in three related tissues in individuals from two geographically separated populations plus F1 individuals to study both tissue and allele specific effects on gene expression. The authors find a large number of differentially expressed genes between tissues and backgrounds and that tissue specific effects were larger than background effects. This finding provides insight into the role of cis and trans effects in differential gene expression. The main takeaway is that there is a lot of variability between tissues and background in terms of genes that show ASE and gene regulation. The regulatory variation vs tau comparison is quite interesting.

The methods used in the paper are straightforward and have been used in many other similar analyses.

Comments:

Figure1: the color scheme used in this figure makes it difficult to distinguish some of the genotypes, in particular the two ZI genotypes from the SU58xZI crosses. Please make these colors more distinct.

Response 1: Thank you for pointing out this potential issue for readers. We have changed the color scheme of Figure 1 as well as Figures 7, S10 and S11, which share the same color scheme, to colors with a stronger contrast. Please see also Response 5 to Reviewer 2.

Figure 2 B: What is the overlap between the gene lists summarized in this table? How much sharing is there between the lists of DE genes between comparisons of the same type with respect to population or the F1s?

Response 2: This is a good question. In order to address it, we examined the overlap in genes detected as differentially expressed (DE) within genotypes among tissues and genotypes for all genotypes for which we had data in all examined tissues. To further address whether the overlapping DE genes showed consistent changes among tissues, we also examined if these genes were consistently upregulated in the same tissue. 50–58% of the genes shown in Fig 2B were differentially expressed in all pairwise tissue comparisons within a genotype, 1,243–1,594 of which were consistently upregulated in the same tissue. The number of overlapping DE genes was similar among genotypes regardless of the genetic background (i.e. genotypes with a shared population or parent did not appear to share more DE genes), with 1,619–1,880 genes being shared among at least two genotypes. We detected 1,045 genes as differentially expressed among all tissues within all genotypes, with 700–924 genes consistently up-regulated in the same tissue. These results can be found in the new S1 Table. All other Supplementary Tables have been renumbered accordingly. We have also revised our Results section to reflect these findings (page 6, lines 172–7 in the current manuscript).

The microbiome composition is a nice addition and while I agree that removing Wolbachia seems reasonable in general I have concerns about how this affects the analysis. Is the difference in % Wolbachia significant between either the Sweden and Zambian populations or either parent and the F1 for the midgut?

Response 3: For completeness we have also addressed this question in the hindgut. The relative abundance of *Wolbachia* was not significantly different among any of the genotypes in the hindgut. Please see Fig R1 and Tables R1 and R2 below. When we performed pairwise comparisons between parental populations and F1 hybrids, the relative abundance of *Wolbachia* only significantly differed in the midgut between the parental Zambian strains and the F1 hybrids (Fig R1, Table R1); however, there was no significant overall effect of genotype alone on *Wolbachia* abundance in our lmer model (Table R2), suggesting that, in general, the exclusion of *Wolbachia* should not affect our conclusions from these analyses. Moreover, when we included *Wolbachia* in these analyses, our results remained qualitatively similar (please see Figs S9 and S10 and Tables S11–S13, formerly Tables S8–S10). We have, therefore, left the analyses in which *Wolbachia* was removed in the main text of the manuscript. We have revised our Results section to reflect that we did not detect any pattern of relative *Wolbachia* abundance among genotypes (page 15, lines 469–71) and have added Table R2 as Table S14 in our Supplementary material. All other Supplemental Tables were renumbered accordingly.

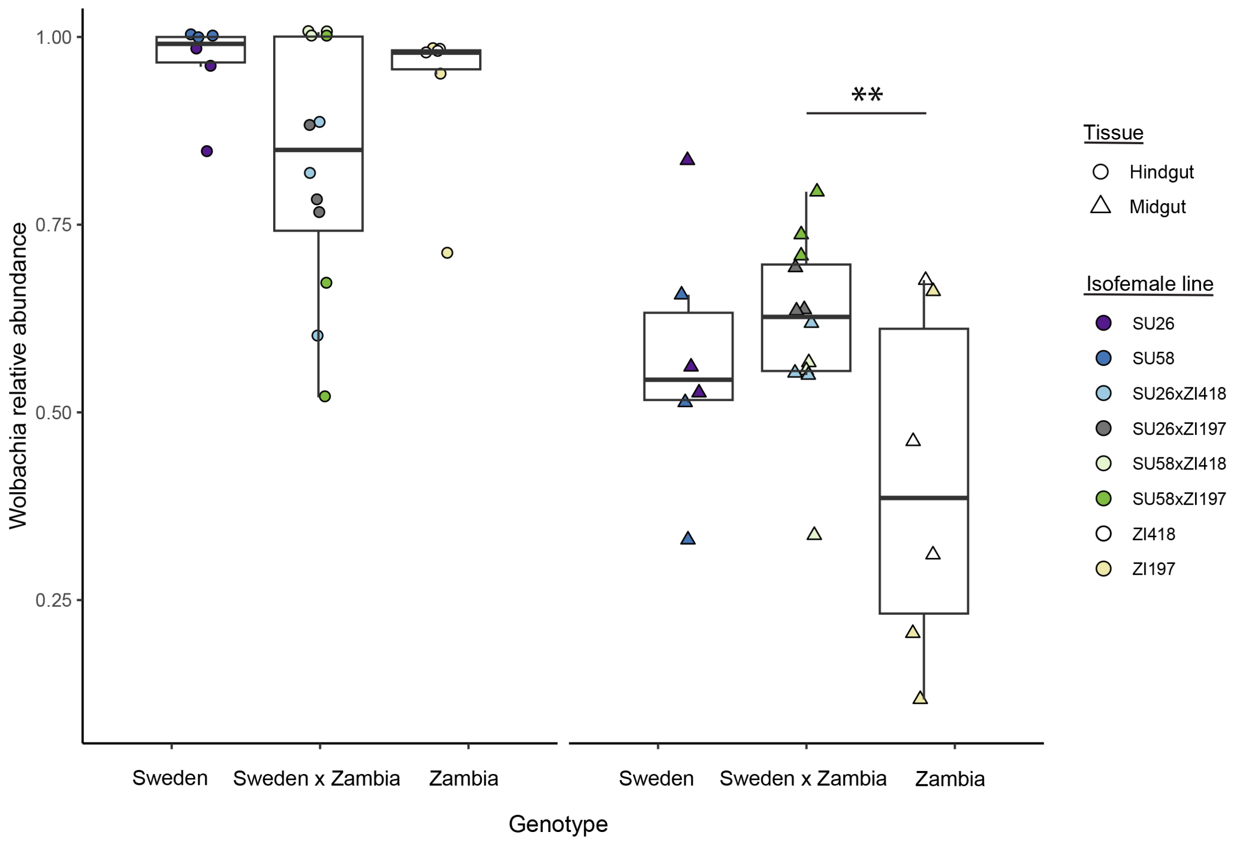

Fig R1: Relative abundance of *Wolbachia* in the midgut and hindgut. ** indicates significant differences of the Shannon index between groups (P = 0.01).

Table R1: Pairwise comparisons of parental strains and F1 hybrids within the midgut and hindgut. The comparisons were performed following the Tukey method. Benjamini-Hochberg-corrected P-values are shown, with significant values indicated in bold.

		**Migdut**			**Hingdut**		
		Sweden	Sweden x Zambia	Zambia	Sweden	Sweden x Zambia	Zambia
**Migdut**	Sweden						
	Sweden x Zambia	0.580					
	Zambia	0.078	**0.01**				
**Hingdut**	Sweden						
	Sweden x Zambia				0.085		
	Zambia				0.687	0.192	

Table R2: Results of lmer comparing the relative abundance of *Wolbachia* in the bacterial communities.

Factor	DF	Chi Sq	*P*
genotype	2	2.801	0.246
tissue	1	62.249	**<0.001**
genotype * tissue	2	9.817	**0.007**

Number of cell types in each tissue - this is mentioned briefly in the discussion, but I think a bit more context about the tissues would be appropriate here. Additional information about the number of cell types known in each of these tissues would help to give context to the findings in the paper.

Response 4: As the reviewer suggests, we have added the current estimates for the number of distinct cell types within each of the examined tissues (page 17, lines 537–9 in the current manuscript) as well as the associated references. However, we should note that recent studies using scRNA-seq data have revealed many previously unknown cell subtypes in the midgut, thus, it is possible that additional subtypes may be identified in the other two examined tissues in the future as more such in depth scRNA-seq datasets become available.

The authors point out that there are differences in the inversion status between some of the lines, but do not address if there is any effect of the inversion in the regulatory variation. Is there, for example, an increase in trans effects within the In(2L)t region for comparisons involving SU26?

Response 5: The reviewer is correct that in the previous version of the manuscript, we mentioned the inversion status of the examined strains but did not examine or mention how variation in inversion status might affect our results. Two inversions are present in our dataset, the *In(2L)*t in SU26 and the *In(3R)K* in ZI197. Previous work in the Malpighian tubule found that although genes affected by the *In(2L)t* inversion make a minor contribution to expression variation in our Malpighian tubule data, the presence of *In(2L)t* could not explain the patterns of dominance or expression observed for backgrounds containing SU26 (Glaser-Schmitt et al 2018; reference 26 in our manuscript). However, we failed to mention this information in our previous manuscript and have now mentioned it in our revised Results section (page 13, lines 409–12).

To address how the presence of these inversions potentially affects regulatory variation, we tested for a significant over- or underrepresentation of genes differentially expressed or displaying ASE between parental strains among genes located within these inversions using a χ^2 test. We focused our analysis of regulatory variation specifically on genes displaying ASE for two reasons: i) If genes located within an inversion are differentially affected by regulatory variation, it is most likely to be variation also located within the inversion, i.e. *cis*-regulatory variation. ii) Genes displaying ASE (Table 1 in our manuscript) are the most inclusive category of *cis*-regulation within our dataset, as they include genes displaying any detectable level of *cis*-regulatory variation, which allowed us to

include as many genes in the analysis as possible. For genes displaying ASE, we did not find any over- or underrepresentation of genes located within either inversion (*P* > 0.29). The only exception was the SU58xZI418 genetic background in the Malpighian tubule, which was significantly enriched for genes located on the *In(2L)t* (*P* = 0.003), despite having neither inversion. Thus, the presence of the *In(2L)t* and *In(3R)K* inversions did not appear to explain the regulatory patterns that we detected. For completeness, we also examined the effect of these inversions on differential gene expression. Genes differentially expressed between parental strains were significantly enriched for genes located within both inversions for all comparisons, including comparisons between strains that did not contain the focal or any inversion ($P < 10^{-15}$ for all), which suggests that the 2L and 3R chromosome arms themselves are enriched for differentially expressed genes, rather than the inversions. These results can be found in the new Table S9. All other Supplemental Tables have been renumbered accordingly. We also have updated our Methods and Results sections to reflect these findings (pages 13–14, lines 405–26; page 20, lines 634–7).

Reviewer #2: In this study, Glaser-Schmitt et al. examine variation in transcript abundance in three tissues (two functionally related ones and one somewhat less related) and in four genetic backgrounds of D. melanogaster to elucidate the evolution of regulatory architecture. To evaluate potential interactions between gene expression variation and microbiome communities, the authors also characterize bacterial composition in the two gut tissues. In both transcriptome and microbiome data, the authors detected pervasive genetic background- and tissue-specific effects, with the latter being typically stronger than the former. Analyses of allele specific expression (ASE) showed that genes with trans-effects are the most abundant regulatory class among genes with non-ambiguous regulatory variation. Not surprising, tissue specificity was higher for both cis- and trans-regulated genes than for genes with conserved expression across all genetic backgrounds. However, genes with trans-effects were more tissue-specific than genes with cis-effects. This observation suggests that variation in gene expression due to trans-regulatory elements can be spatially fine-tuned as well as (or even better than) that regulated by cis elements.

This is a well-written manuscript based on mRNAseq data analysis. The analyses are sound, and the figures of the manuscript are elaborated and successfully encapsulate the main points of the study in a clear and informative way, which is remarkable when we consider the large amount of new data reported. The results have a large descriptive component and yet they are interesting and exciting. This study fills a current gap in the knowledge of the field and will spark new research on the genetic basis of regulatory evolution. I don't have any major issues, so I am recommending acceptance for publication in PLoS Genetics with minor revisions. I have just a few minor points and some editorial comments (see below).

1. The term dominance (h) divergence is used to describe variation in degree of dominance among genotypes (within the same tissue) but also among tissues (within the same genotype). It is not clear, however, how dominance divergence is calculated. Furthermore, the word divergence is usually reserved for differences between species but in this study, it also refers to differences among tissues so, in my view, it is a bit confusing. Perhaps the authors would consider alternative terms. "Dominance distance"? Along these lines, what is the difference between "Dominance" and "Magnitude Dominance" (Fig. 4)? Additional clarification is needed.

Response 1: Thank you for pointing out these potential issues for readers. We calculated divergence in dominance *h* between two genetic backgrounds or tissues as 1 – Spearman's ρ between the two. While we briefly mentioned this in the Results section text (page 11, line 266 in

our previous manuscript), we failed to include how divergence was calculated in our Methods section. We have now included an explanation of how dominance divergence was calculated in the Figure 3 legend as well as the methods section (page 8, line 247; page 24, lines 743–6 in the current manuscript). Similarly, we have also now included an explanation of how expression divergence was calculated in the methods section (page 23, line 722–4 in the current manuscript), which was missing from our previous manuscript.

In Figure 4, "dominance" refers to phenotypic dominance *h*, which can have positive or negative values and reflects which genetic background is more dominant. "Magnitude of dominance" refers to the absolute value of dominance *h,* which can only have positive values and allows us to directly compare the magnitude of the degree of dominance regardless of which genetic background is more dominant. We have made this distinction more clear in the revised Figure 4 legend and Results section text (page 11, line 342; page 12, lines 358–9 in the current manuscript).

While the term "divergence" is often applied to divergence between species, it can describe differentiation at all organismal levels from cells to species, including differentiation between populations or tissues. For just a few examples in the literature, please see Cooper et al (2020 in Molecular Ecology), Schlebusch et al (2020 in Molecular Biology and Evolution), Johnson and Kelly (2020 in Evolutionary Applications), Reboud et al (2023 in Genome Biology and Evolution), Glazko and Mushegian (2010 in Biology Direct), Jin et al (2021 in Nature Communications). Moreover, because we calculate divergence in the same way for expression and dominance as well as for among tissues and genetic backgrounds, we wanted to use consistent terminology regardless of the organismal level we were comparing. We have therefore chosen to keep the current terminology; however, in order to make our terminology more clear and prevent potential reader confusion, we have made our usage of the term "divergence" more clear in the Results and Discussion sections (page 6, lines 162–3; page 9, lines 269–71; page 18, lines 572–4 in the current manuscript). Please see also Respose 2 to Reviewer 2 below.

2. Following the previous point, the terms "gene expression divergence" are used to refer to differences in gene expression among tissues. However, "gene expression divergence" has been extensively used to describe differences in gene expression between species so it would be better to find an alternative. Maybe "gene expression distance?" Not an easy task.

Response 2: Please see also Respose 1 to Reviewer 2 above. The reviewer is correct that "gene expression divergence" is often used to decribe differences in expression between species; however, it is applicable across all levels of organismal organization where differentiation occurs, including tissues. Indeed, a recent paper in Nature Communications (Jin et al 2021) about the pig BodyMap transcriptome used it in the context of differentiation between tissues within a species as well as differentiation between species. In order to prevent potential reader confusion, we have better clarified our use of the term in the text (page 6, lines 162–3; page 9, lines 269–71; page 18, lines 572–4).

3. This study focuses on gene expression in females. Is there sexually dimorphic gene expression in the 3 tissues examined in D. melanogaster? How much? I wonder about the relative contribution of genes with different degrees of sex-biased expression to the main findings of the study. One could potentially analyze male-, female- and nonsex-biased genes separately, and see to what extent conclusions hold. This may be relevant, considering that the different categories of sex-biased expression genes have been shown to have, for instance, different rates of protein

evolution. The downside to this approach is perhaps the potential loss of statistical power. Just a thought for the authors to consider.

Response 3: This is an interesting question, but, as the reviewer points out, we are unable to fully answer it with our current dataset as we do not have male expression data with which to identify female-, male-, and unbiased genes in the examined strains and their F1 hybrids. We therefore used the male and female FPKM data available for these tissues from Flyatlas2 to calculate sex bias in the midgut, hindgut, and Malpighian tubules. Within a given tissue and genotype combination, 4.75–41.64% of analyzed genes displayed sex-biased gene expression. The midgut had the most genes with sex-biased gene expression with ~40% of genes detected as having sex-biased gene expression, while only 4.75–9.99% of genes displayed sex-biased gene expression in the other examined tissues. The number of genes falling into each sex bias category can now be found in the new Table S8 in our revised manuscript. All other Supplemental Tables have been renumbered accordingly.

Because sexually dimorphic regulatory effects are often in *cis* (Meiklejohn et al 2014) and *cis*-regulatory effects have been found to differ across different levels of sex bias and across tissues/body parts (Puixeu et al 2023), we focused our analysis on genes we detected as displaying allele specific expression (ASE; Table 1 in our manuscript). With the exception of the midgut, we did not detect many genes showing strongly sex-biased gene expression in our dataset (LFC $\geq$ 1.5; 0–17 per genetic background in the hindgut and Malpighian tubule). Although we detected more genes with moderately sex-biased gene expression in these tissues (0.5 $\geq$ LFC $\geq$ 1.5; 59–303), for half of all examined genetic background and tissue combinations, not enough genes were detected as female- and/or male-biased to allow us to make meaningful comparisons among sex bias categories (i.e. fewer than 5 genes were expected to display ASE in at least one sex bias category, which can result in poor χ^2 approximations). Therefore, we categorized genes as either sex-biased or unbiased and tested for an enrichment (or dearth) of sex-biased genes in genes displaying ASE with a χ^2 test.

In the Malpighian tubule, we detected a significant enrichment of sex-biased genes in genes we detected as displaying ASE for both genetic backgrounds (P = 0.0010 for both), and in the hindgut we detected an enrichment of sex-biased genes in all genetic backgrounds (P < 0.02 for all) except SU26xZI418 (P = 0.2929). Conversely, we only detected a significant enrichment of sex-biased genes in the midgut in the SU26xZI197 background (P = 0.0116). Thus, genes displaying ASE were enriched for sex-biased gene expression, however, similar to many of our other findings, this enrichment was dependent upon the tissue and genetic background in which they were detected. We have added a section to our Results describing these results (page 13, lines 383–404) and modified our Methods section (pages 26–27, lines 833–43) to reflect these findings.

4. A technical comment. One of the observations of this study is that divergence for dominance is significantly higher than for gene expression (Bonferroni-corrected P < 10-14), which suggests that phenotypic dominance of expression is much less conserved among tissues and genotypes than expression itself (lines 275-276). What statistical test was used here? Did it consider that gene expression divergence is expected to be distributed between 0 and 1 while dominance divergence between -1 and 1? Please elaborate.

Response 4: Significance was assessed with a *t*-test; however, this was unclear in our previous manuscript. We have now made this more clear in the revised Results and Methods sections (page 9, line 279; page 23, line 724; page 24, lines 745–6). Divergence was calculated in the same

manner for both dominance and gene expression divergence (1 – Spearman's ρ; please see also Response 1 to Reviewer 2), which allows divergence in these traits to be compared on the same scale. However, it does not take into account that the data that goes into the calculation of Spearman's ρ was calculated using different types of data, specifically, mean normalized gene counts for gene expression divergence or the dominance *h* values, which were calculated from mean normalized gene counts for 3 genotypes (the two parental strains and their F1 hybrid), for dominance divergence. We have made the calculation of divergence for both more clear in our Methods section (page 23, line 722–23; page 24, lines 743–5). It is possible that part of the reason for the detected difference between dominance and expression divergence is that expression is measured based on expression in one genotype, while dominance is based on expression in three genotypes. We have now mentioned this as a possibility in our revised Results section (page 9, lines 281–3).

5. RNAseq data. Since poly-A selection was performed using total RNA, it should formally be mRNAseq data instead.

Response 5: As the reviewer suggests, we have changed all instances of "RNA-seq" to "mRNA-seq" in our revised manuscript.

6. Figures are very informative. Fig. 1 & 7; I would suggest a more contrasting pattern of colors for increased clarity. Fig. 4 B; X-axis legend should be "Intersection class", not "Interaction class". Fig. 4C and D; the colors are helpful but I had difficulty seeing the asterisks of statistical significance so I would suggest increasing size a bit.

Response 6: Thanks and thank you for pointing out these potential issues for readers. We have changed the color scheme of Figures 1 and 7 to include colors with a stronger contrast. For consistency, we have also changed the color scheme for Figures S10 and S11. Please see also Response 1 to Reviewer 1. We have also fixed the typo in the Figure 4B X-axis and increased the size of the asterisks indicating significance as the reviewer suggests.

EDITORIAL COMMENTS

• Ln 103 "… single tissues, and/or highly diverged tissues…." replace with "… single tissues, and/or highly functionally diverged tissues…."

Response 1: We have rephrased this sentence as suggested (page 4, line 103 in the current manuscript).

• Ln 132 "… From ASE data, we found that both cis and trans effects were more tissue-specific than genes with no differential expression…" replace with "… From ASE data, we found that genes with both cis and trans effects were more tissue-specific than genes with no differential expression… "

Response 2: We have rephrased this sentence as suggested (page 5, lines 132–4 in the current manuscript).

• Ln 151 " … some overlap between SU58, ZI418 and their F1 hybrid and well as …" replace with " … some overlap between SU58, ZI418 and their F1 hybrids, and as well as …"

Response 3: Thank you for pointing out this typo. We had not intended to include two conjunctions here. We have changed the text to: "some overlap between SU58, ZI418 and their F1 hybrids, as well as…" (page 5, line 152 in the current manuscript).

• Ln 249 "In order to compare the magnitude of dominance regardless of which allele was dominant, we calculated h such that values between 0 and 1 or 0 and -1 represent varying degrees of additivity and dominance with values closer to 1 or -1 being more dominant and -1 representing complete dominance of the Swedish background and 1 representing complete dominance of the Zambian background, while values outside this range represent cases of overdominance of the respective background (see Methods for more details)." replace with "In order to compare the magnitude of dominance regardless of which allele was dominant, we calculated h such that values between 0 and 1 or 0 and -1 represent varying degrees of additivity and dominance, with values closer to -1 representing complete dominance of the Swedish background and 1 representing complete dominance of the Zambian background, while values outside this range represent cases of overdominance of the respective background (see Methods for more details)."

Response 4: We have rephrased this sentence as suggested (page 8–9, lines 253–8 in the current manuscript).

• Ln 351 "… and this was only significant after multiple test correction in the midgut…" replace with "… and this was significant after multiple test correction only in the midgut…"

Response 5: We have rephrased this sentence as suggested (page 12, lines 360–1 in the current manuscript).

• Ln 753 " … the ASE analysis section above in order balance statistical power…" replace with " … the ASE analysis section above in order to balance statistical power…"

Response 6: We have rephrased this sentence as suggested (page 26, lines 818–9 in the current manuscript).
